# ESC-Leitlinie 2020 zur Behandlung von Erwachsenen mit angeborenem Herzfehler (ACHD)

**DOI:** 10.1007/s00059-020-05003-0

**Published:** 2020-12-01

**Authors:** Francisco Javier Ruperti-Repilado, Corina Thomet, Markus Schwerzmann

**Affiliations:** Zentrum für angeborene Herzfehler, Medizinalbereich Herz und Gefässe, Universitätsklinik Inselspital und Universität Bern, Freiburgstraße 15, 3010 Bern, Schweiz

**Keywords:** Herzanomalie, Vererbung, Pulmonalarterielle Hypertonie, Kardiale Arrhythmien, Interventionen, Cardiac anomaly, Heredity, Pulmonary arterial hypertension, Cardiac arrhythmias, Interventions

## Abstract

Im August 2020 veröffentlichte die European Society of Cardiology (ESC) neue Leitlinien zur Behandlung von Erwachsenen mit angeborenem Herzfehler („adult congenital heart disease“, ACHD). Die bisherigen Empfehlungen des Jahres 2010 wurden den Entwicklungen der letzten 10 Jahre in Diagnostik und Therapie angepasst. Nach wie vor entsprechen die Empfehlungen aber nahezu ausschließlich einem Evidenzgrad C (Expertenmeinung oder Erkenntnisse aus kleinen respektive retrospektiven Studien oder Registerstudien). Wir sprechen von einer heterogenen Patientenpopulation mit einer Vielzahl von unterschiedlichen Herzfehlern und Korrektureingriffen, die sich dank sinkender perioperativer Mortalität und weiterer medizinischer Fortschritte in konstantem Wachstum befindet und älter wird. Die aktuellen Leitlinien sind dementsprechend nicht nur auf die akute Behandlung kardialer Probleme fokussiert, sondern legen das Augenmerk auf eine gesamtheitliche longitudinale Betreuung. Ergänzt werden diese allgemeinen Aspekte durch defektspezifische Empfehlungen, wobei v. a. Fortschritte bei Arrhythmiediagnose und -behandlung, invasiver Kardiologie sowie pulmonalarterieller Hypertonie zu wesentlichen Anpassungen führten. Erstmalig wird in den Leitlinien 2020 auch die Thematik von Koronaranomalien aufgegriffen.

## Abstract (en)

The new guidelines of the European Society of Cardiology (ESC) on treatment of adult congenital heart disease (ACHD) were published in August 2020. The previous recommendations from 2010 were adapted to reflect the diagnostic and therapeutic progress made in the past 10 years. The recommendations are nearly exclusively based on an evidence level C (consensus of opinion of experts or knowledge from small studies, retrospective studies or registries). This is not surprising considering the heterogeneous patient population with a multitude of cardiac defects and repair strategies performed in the past. The cohort of ACHD patients is steadily growing in numbers and is becoming older due to reduced perioperative morbidity and mortality and further medical progress. Therefore, the current guidelines do not focus solely on the acute treatment of cardiac problems but also address the importance of a comprehensive longitudinal follow-up for a chronic, lifelong disorder. On a defect-specific level, progress in the past decade in arrhythmia diagnosis and management, percutaneous interventions and the treatment of pulmonary arterial hypertension have led to many revised or new recommendations. Finally, the 2020 guidelines also address for the first time the management of coronary anomalies.

## Einleitung und Namensgebung

10 Jahre nach Veröffentlichung der „Guidelines for the management of grown-up congenital heart disease“ [1] der European Society of Cardiology (ESC) wurde 2020 eine revidierte Fassung publiziert [2]. Die überarbeitete Leitlinie gliedert sich in allgemeine und herzfehlerspezifische Abschnitte. Der allgemeine Teil bildet die Tatsache ab, dass es sich bei angeborenen Herzfehlern in der Mehrzahl der Fälle um eine chronische Erkrankung handelt. Dies erklärt die Wichtigkeit einer lebenslangen kardiologischen Betreuung inklusive der Notwendigkeit eines strukturierten Transitionsprozesses. Junge Erwachsene mit angeborenen Herzfehlern müssen ihre Gesundheit losgelöst von der elterlichen Fürsorge in die eigenen Hände nehmen. Um einem gesamtheitlichen Ansatz gerecht zu werden, wurden die Personalempfehlungen für ein spezialisiertes ACHD(„adult congenital heart disease“)-Zentrum überarbeitet (Tab. 1). Hierzu ist ein multiprofessionelles Behandlungsteam nötig.

**Table 1:** 

Teammitglieder	Empfohlene Anzahl
Kardiologe mit ACHD-Qualifikation	≥2
Imaging-Spezialist mit ACHD-Expertise (zertifiziert TTE/TEE, MRT, CT)	≥2
Kongenitaler interventioneller Kardiologe	≥2
Herzchirurg mit Expertise in angeborenen Herzfehlern	≥2
Anästhesist mit Expertise in angeborenen Herzfehlern	≥2
Spezialisierte Pflegefachperson (falls nationale Richtlinien dies zulassen)	≥2
Elektrophysiologe mit ACHD-Expertise	≥1
Experte für pulmonale Hypertonie	≥1
Klinischer Genetiker	≥1
Psychologe	≥1
Sozialarbeiter	≥1
Palliative Care Team	1

*CT* Computertomographie, *MRT* Magnetresonanztomographie, *TEE* transösophageale Echokardiographie, *TTE* transthorakale Echokardiographie

Der Grundsatz der lebenslangen Betreuung und der älter werdenden Patientengruppe spiegelt sich auch in der neuen Namensgebung der Leitlinie wieder: während der bisherige Name „GUCH“ („grown-up congenital heart disease“) sich auf eine erwachsen werdende Patientengruppe bezog, wird neu mit „ACHD“ ausgedrückt, dass es sich um erwachsene Patienten mit altersbedingten Komplikationen und zusätzlich erworbenen Erkrankungen handelt.

## Allgemeine Empfehlungen

Erreichen Jugendliche mit angeborenem Herzfehler das Erwachsenenalter, wird der Transfer, d. h. der Wechsel der Betreuung vom kinderkardiologischen zum Behandlungsteam für Erwachsene, empfohlen [3]. Ein solcher Transfer sollte in einen Transitionsprozess mit einer ihm vorausgehenden strukturierten Vorbereitungsphase eingebettet sein. Entsprechend den Bedürfnissen des Patienten sollte nach dem Transfer weitere edukative und soziale Unterstützung angeboten werden. Hierzu braucht es eine Zusammenarbeit zwischen dem Kinderkardiologie- und dem ACHD-Team. Dazu gibt es eine für die Transition verantwortliche Person, welche den Prozess koordiniert und begleitet [4].

Dank der verbesserten medizinischen Betreuung der letzten Jahrzehnte erreichen mehr und mehr ACHD-Patienten auch ein höheres Alter, wobei der Zuwachs in der Patientengruppe mit hoch komplexen Herzfehlern prozentual am höchsten ist [5]. Sie stellen eine neue Herausforderung ans Gesundheitssystem dar, nicht nur aufgrund ihrer Komplexität, sondern auch aufgrund ihres Ressourcenbedarfs [6]. Um vermeidbare erworbene Komorbiditäten so weit wie möglich zu verhindern, müssen präventive Maßnahmen (Bewegung, Ernährung etc.) bereits im Kindes- und Adoleszentenalter angesprochen und eingeleitet werden.

Unabhängig von der Komplexität des Herzfehlers wünschen ACHD-Patienten, mit ihrem Behandlungsteam über Lebenserwartung, medizinische Möglichkeiten und Spätkomplikationen zu sprechen [7]. Um diesem Wunsch Rechnung zu tragen, wird ein strukturiertes Advance-Care-Planning-Gespräch empfohlen. Detaillierte Informationen zu diesem Thema finden sich in der Leitlinie „Recommendations for advance care planning in adults with congenital heart disease“ [8].

## Arrhythmien

**Figure 1:**
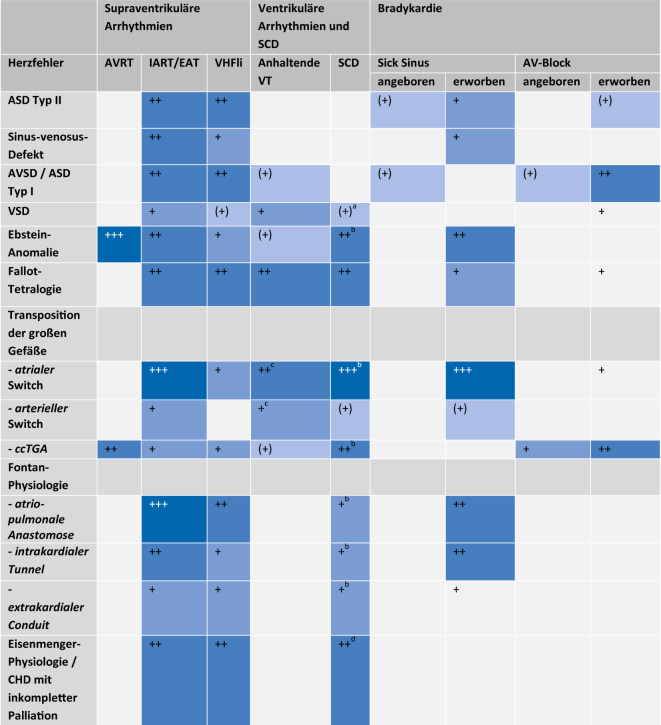

Bei Patienten mit angeborenen Herzfehlern kann das gesamte Spektrum von Rhythmusstörungen vertreten sein. Die Häufigkeit einzelner Arrhythmien ist in Abb. 1 zusammengefasst. Diesbezüglich wurden 2018 in einem ESC-Positionspapier [9] erstmals Behandlungsempfehlungen zu Rhythmusstörungen bei ACHD formuliert, die sich nun in den aktuellen Leitlinien wiederfinden. Im Abschnitt des Dokuments über Herzfehler wird je nach Vitium das Arrhythmiemanagement erwähnt.

Exemplarisch wurden bei der korrigierten Fallot-Tetralogie in den letzten Jahren die anatomischen Voraussetzungen für Kammertachykardien („ventricular tachycardia“, VT) detailliert untersucht und publiziert [10, 11]. Bei diesem Vitium wird empfohlen, im Vorfeld oder während eines Pulmonalklappenersatzes bei Patienten mit symptomatischen anhaltenden VT gezielt den kritischen Isthmus zu identifizieren und zu abladieren, da postinterventionell die Ablationsmöglichkeiten durch die Melody-Klappe oder einen chirurgisch implantierten Conduit erschwert bis verunmöglicht sein können. Unter gewissen Voraussetzungen (normale biventrikuläre Pumpfunktion, fehlende Induzierbarkeit und etablierter Leitungsblock) kann nach erfolgreicher Ablation auf eine zusätzliche ICD(implantierbarer Kardioverter-Defibrillator)-Implantation verzichtet werden.

Bei allen elektrophysiologischen Interventionen ist die Expertise der Rhythmologen für ACHD wichtig. Es ist ein multidisziplinäres und erfahrenes Team nötig, um ein optimales Ergebnis erzielen zu können. Aus diesem Grunde wird bei Patienten mit mäßig oder hoch komplexen Herzfehlern und Arrhythmien geraten, nötige Untersuchungen in einem ausgewiesenen ACHD-Referenzzentrum durchführen zu lassen.

## Pulmonale Hypertonie

Die pulmonale Hypertonie (PH) ist auch heute noch ein prognostisch wichtiger Faktor bei angeborenen Herzfehlern. Seit den GUCH-Leitlinien 2010 haben sich die medikamentösen Möglichkeiten in der Behandlung der pulmonalarteriellen Hypertonie (PAH) weiterentwickelt. Im Jahr 2018 wurde zudem die Definition der PH geändert (Tab. 2). Die Leitlinien von 2020 zur ACHD nehmen diese Entwicklung auf. Bei der medikamentösen Therapie der PAH lehnen sie sich an die aktuellen ESC-PAH-Leitlinien von 2015 [12] an und empfehlen eine proaktivere Strategie. Wie bei der idiopathischen PAH kommt auch bei der shuntinduzierten PAH der Risikostratifizierung eine wichtige Rolle zu, da sie Einfluss auf die medikamentöse PAH-Therapie hat. Bei der Subgruppe von Patienten mit residueller PAH nach Shuntkorrektur wird eine Kombinationstherapie vorgeschlagen, die bei Patienten mit ungünstigen prognostischen Faktoren von Beginn an eine parentale Verabreichung von Prostaglandinen beinhaltet.

**Table 2:** 

Definition	Hämodynamik	Klinisches Beispiel
Pulmonale Hypertonie	Mittlerer PA-Druck >20 mm Hg	Alle
Präkapilläre PH (PAH)	Mittlerer PA-Druck >20 mm Hg Mittlerer PAWP ≤15 mm Hg PVR ≥3 WU	Shuntdefekte vor und nach Korrektur (inklusive Eisenmenger-Syndrom) Komplexe Herzfehler (inkl. univentrikuläres Herz und segmentale PAH)
Isolierte postkapilläre PH	Mittlerer PA-Druck >20 mm Hg Mittlerer PAWP >15 mm Hg PVR <3 WU	Dysfunktion des Systemventrikels Dysfunktion der systemischen AV-Klappe Lungenvenenobstruktion Cor triatriatum
Kombinierte post- und präkapilläre PH	Mittlerer PA-Druck >20 mm Hg Mittlerer PAWP >15 mm Hg PVR ≥3 WU	Siehe isoliert postkapilläre PH Siehe isoliert postkapilläre PH, in Kombination mit Shunt-Defekten/komplexen Herzfehlern

*AV* atrioventrikulär, *PA* pulmonalarteriell, *PAWP* pulmonaler Wedge-Druck, *PAH* pulmonalarterielle Hypertonie, *PH* pulmonale Hypertonie, *PVR* Lungenwiderstand, *WU* Wood Units

Bei Patienten mit irreversibler shuntinduzierter PAH (inkl. Eisenmenger-Syndrom) steht weiterhin eine Monotherapie mit Endothelinrezeptorantagonisten im Vordergrund. Falls keine klinische Verbesserung eintritt, sollte eine Kombinationstherapie angewandt werden. Explizit wird darauf hingewiesen, dass auch bei Patienten mit Eisenmenger-Syndrom nötigenfalls parenterale Prostaglandine eingesetzt werden sollten, doch verzugsweise in inhalativer oder subkutaner Form, da bei der kontinuierlichen intravenösen Verabreichung das Risiko paradoxer Embolien besteht.

**Figure 2:**
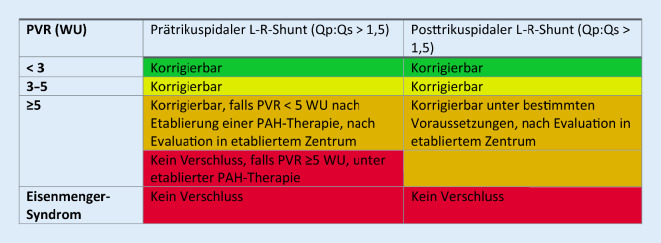

Die neuen Leitlinien nehmen ebenfalls Stellung zur Frage, bis wann ein Shuntverschluss bei bereits etablierter PH möglich ist, und schlagen ein differenzierteres Bild als die ESC-PAH-Leitlinien vor. Im Vordergrund steht die Erfahrung, dass bei posttrikuspidalem Shunt (z. B. Ventrikelseptumdefekt, VSD) höhere pulmonale Widerstände einen Shuntverschluss unter bestimmten Umständen noch erlauben, als dies beim prätrikuspidalen Shunt (z. B. Vorhofseptumdefekt [„atrial septal defect“, ASD]) möglich ist. Diese Einschätzung basiert auf Expertenmeinung. Unbestritten ist die Tatsache, dass ein Defektverschluss bei fortgeschrittener PAH unterlassen werden sollte, da der klinische Verlauf nach Defektverschluss bei schwerer PAH ungünstiger ist als bei der shuntinduzierten PAH inklusive Eisenmenger-Syndrom [13]. Dies erklärt die Wichtigkeit einer kompletten hämodynamischen Evaluation mittels Herzkatheter vor einem Shuntverschluss, sobald die nicht-invasive Diagnostik eine PH nahelegt. Konkret bedeutet dies, dass eine vollständige invasive Abklärung vor einer Intervention empfohlen wird, falls der echokardiographisch geschätzte systolische PA-Druck mehr als 40 mm Hg beträgt oder indirekte Zeichen einer PH vorliegen. Abb. 2 fasst die aktuellen Empfehlungen bezüglich Shuntverschluss zusammen. Wichtig ist auch, dass die Entscheidung, bis wann ein Defekt verschlossen werden kann, nicht nur vom pulmonalen Widerstand abhängt, sondern vom gesamten klinischen Bild.

## Vorhofseptumdefekt

**Figure 3:**
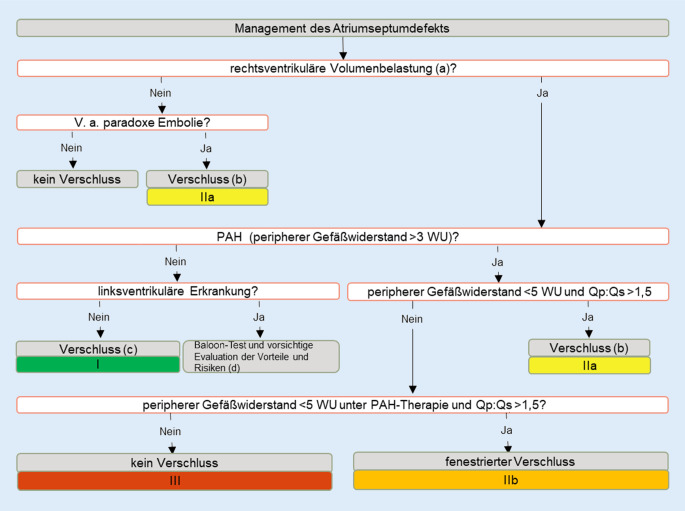

Der ASD ist einer der wenigen Herzfehler, die im Kindesalter symptomarm sein können und deswegen bei Erwachsenen nicht immer bereits frühzeitig entdeckt wurden. Entsprechend ist die Diagnose im Erwachsenenalter keine Seltenheit. Die Leitlinien 2020 weisen darauf hin, dass der Verschluss eines ASD auch beim älteren Patienten die Symptome der Atemnot und der eingeschränkten Leistungsfähigkeit lindert. Ein ASD vom Primumtyp kann nur chirurgisch, ein ASD vom Sekundumtyp oft perkutan verschlossen werden. Es wird empfohlen, bei Patienten mit fortgeschrittener diastolischer oder systolischer Linksherzdysfunktion vor dem definitiven Verschluss eine Probeokklusion durchzuführen und anhand des Verhaltens der Füllungsdrücke nach Okklusion zu entscheiden, ob ein vollständiger Verschluss erfolgen, ein fenestriertes Device zum Einsatz kommen oder gar kein Verschluss unternommen werden soll. Abb. 3 zeigt den vollständigen Algorithmus hinsichtlich eines ASD-Verschlusses. Patienten mit residuellem Shunt, dokumentierten Arrhythmien vor oder nach Intervention, pulmonaler Hypertonie oder einem ASD-Verschluss nach dem 40. Lebensjahr benötigen regelmäßige Nachkontrollen. Auch beim ASD-Verschluss mittels Device-Implantation wird eine längerfristige, altersunabhängige Nachbetreuung alle 3 bis 5 Jahre als sinnvoll erachtet.

## Obstruktionen des linksventrikulären Ausflusstrakts, Aortopathie bei bikuspider Klappe

Die Behandlung der Obstruktionen des linksventrikulären Ausflusstrakts („left ventricular outflow tract obstruction“, LVOTO) unterscheidet sich in Abhängigkeit von der Lokalisation der Flussbehinderung (subvalvulär, valvulär oder supravalvulär). Die sub- oder supravalvuläre Obstruktion kann in aller Regel ohne Klappenersatz behoben werden. Bei der valvulären LVOTO ist die bikuspide Aortenklappe der häufigste Grund für eine Klappenstenose.

Zu beachten ist die Assoziation der bikuspiden Aortenklappe mit der Dilatation der Aorta ascendens. Aktuell stimmen die Behandlungsindikationen zum Ascendensersatz mit den ESC-Empfehlungen zur Aortopathie von 2014 überein [14]. Ab einem Diameter von 55 mm sollte die Aorta ascendens bei allen Patienten mit bikuspider Klappe ersetzt werden, ab 50 mm bei Patienten mit zusätzlichen Risikofaktoren (Aortenisthmusstenose, hypertensive Blutdruckwerte, Aortendissektion in der Familienanamnese, Progression der Aortendiameter >3 mm/Jahr) und ab 45 mm auch bei Patienten, die sich einer Aortenklappenoperation unterziehen müssen.

Meistens ist die Therapie der Aortenstenose der Klappenersatz. Die Ballonvalvuloplastie ist nur sehr selten eine Behandlungsoption, z. B. bei jungen Erwachsenen mit nichtkalzifizierten Klappen. Die Indikationen zum Eingriff orientieren sich an den ESC-Herzklappenleitlinien von 2017 [15]. Entsprechend wurde die Definition der schweren Aortenklappenstenose angepasst und der mittlere Gradient von 50 mm Hg oder mehr auf 40 mm Hg oder mehr bei erhaltenem Herzzeitvolumen gesenkt. Beim asymptomatischen Patienten mit schwerer Aortenstenose werden neben der Linksherzdysfunktion (Ejektionsfraktion [EF] < 50 %) und dem Blutdruckabfall unter Belastung nun auch das chirurgische Risiko, der Spitzengradient über der Obstruktion (>5,5 m/s), die BNP(„brain natriuretic peptide“)-Bestimmung sowie der Schweregrad einer PH zur Indikationsstellung des Klappenersatzes berücksichtigt. Bei einem BNP-Wert, der das 3‑Fache der altersentsprechenden Norm übersteigt, oder bei einem systolischen PA-Druck von mehr als 60 mm Hg kann ein Eingriff bei niedrigem Risiko in Betracht gezogen werden.

Entsprechend der Definition einer schweren valvulären Obstruktion werden die Grenzwerte der schweren supra- und subvalvulären Obstruktion auch auf einen mittleren Gradienten von 40 mm Hg oder mehr gesenkt. Bei der supravalvulären Obstruktion gilt es zu beachten, dass der echokardiographisch gemessene Gradient nicht immer dem effektiven Druckabfall über der Obstruktion entspricht. Die physikalischen Voraussetzungen, die zur Anwendung der vereinfachten Bernoulli-Gleichung erfüllt sein müssen, sind je nach Ausdehnung der Obstruktion und Größe der Aorta ascendens nicht gegeben. Im Zweifelsfall ist eine invasive Abklärung nötig.

Bei der Subaortenstenose gilt es, die mögliche Entwicklung einer schweren Aortenklappeninsuffizienz zu berücksichtigen. Falls ausschließlich eine Stenose vorliegt, decken sich die Indikationen zum Eingriff mit Empfehlungen bei supravalvulärer Obstruktion. Falls sich als Folge der Subaortenstenose eine schwere Aorteninsuffizienz entwickelt hat, orientieren sich die Behandlungsindikationen an den ESC-Herzklappenleitlinien von 2017. Eine weitere Indikation zur Resektion der Obstruktion bleibt weiterhin die Progression der Aorteninsuffizienz auf eine mindestens mittelschwere Insuffizienz. Hier geht es darum, durch einen frühzeitigen chirurgischen Eingriff einen späteren Klappenersatz, wenn möglich, zu vermeiden.

## Aortenisthmusstenose

Für die Empfehlung zur Intervention beim Erwachsenen mit nativer oder residueller Aortenisthmusstenose ist das Vorliegen einer proximalen arteriellen Hypertonie ausschlaggebend. Falls die proximalen Blutdruckwerte, die typischerweise am rechten Arm gemessen werden, im hypertensiven Bereich liegen und ein invasiv bestätigter Spitzengradient von mehr als 20 mm Hg über der Obstruktion vorliegt, ist eine Intervention indiziert, wobei ein perkutanes Vorgehen favorisiert wird. Neu wird auch beim normotensiven Patienten mit einem invasiven Spitzengradienten von mehr als 20 mm Hg über der Obstruktion ein Kathetereingriff empfohlen, sofern er technisch möglich ist. Weiterhin kann beim normotensiven Patienten mit einer mindestens 50%igen Einengung im Isthmusbereich relativ zum Aortendiameter auf Zwerchfellhöhe unabhängig vom Spitzengradienten eine Intervention in Betracht gezogen werden. Neuerdings wird die invasive Messung zur Indikationsstellung herangezogen und nicht mehr allein der nicht-invasiv gemessene Arm-Bein-Blutdruckgradient verwendet. Der echokardiographisch gemessene Gradient über der Aortenisthmusstenose spiegelt oft nicht den effektiven Gradienten wider. Die Leitlinien betonen zusätzlich den Stellenwert der 24-Stunden-Blutdruckmessung zur Diagnose der arteriellen Hypertonie. Die medikamentöse Behandlung einer proximalen oder residuellen Hypertonie richtet sich nach den ESC/ESH(European Society of Hypertension)-Empfehlungen 2018 [16].

## Obstruktionen des rechtsventrikulären Ausflusstrakts (RVOTO)

Das Behandlungskonzept der Obstruktionen des rechtsventrikulären Ausflusstrakts („right ventricular outflow tract obstruction“, RVOTO) außerhalb der Fallot-Tetralogie (ob nun subvalvulär, valvulär, supravalvulär oder kombiniert) richtet sich zunächst nach dem Schweregrad der Obstruktion. Anschließend stellt sich die Frage, ob ein Klappenersatz nötig ist. Als drittes Kriterium werden Symptome und Klinik berücksichtigt. Wenn immer möglich, sollte ein Klappenersatz vermieden werden. Lässt die Anatomie nur dieses zu, wird aufgrund der Langzeitrisiken (z. B. Endokarditis, Reinterventionen) ein Eingriff nur beim symptomatischen Patienten oder bei Patienten, deren Funktion des rechten Ventrikels über die Zeit abnimmt, bzw. wenn die Druckbelastung für den Ventrikel sehr hoch ist (>80 mm Hg) in Erwägung gezogen. Ist hingegen die Obstruktion mittels Ballonvalvuloplastie oder Geweberesektion behebbar, wird auch beim asymptomatischen Patienten eine Intervention empfohlen, sobald der RVOTO-Spitzengradient über 64 mm Hg liegt. Auch bei der RVOTO-Quantifizierung mittels Echo muss beachtet werden, dass nur bei der singulären diskreten Stenose eine zuverlässige Korrelation zwischen der gemessenen Flussgeschwindigkeit und dem effektiven Druckgradienten besteht.

## Fallot-Tetralogie

Die chirurgische Korrektur der Fallot-Tetralogie hat sich im Laufe der Zeit gewandelt. Vom Ziel einer radikalen Behebung der Obstruktion mittels großzügiger Muskelresektion und falls nötig einer transannulären Patch-Erweiterung wird heute, wenn möglich, Abstand genommen. So wird der Erwachsene mit korrigierter Fallot-Tetralogie, nativem Ausflusstrakt und schwerer Pulmonalinsuffizienz immer seltener angetroffen. Dennoch stellt die schwere Pulmonalinsuffizienz, auch als Folge einer Degeneration eines implantierten klappentragenden Conduits vom rechten Ventrikel zum Pulmonalarterie (RV-PA-Conduit), weiterhin eine Herausforderung dar. Die Leitlinien spiegeln die Entwicklung des perkutanen Pulmonalklappenersatzes wider und gewichten die Volumina des rechten Ventrikels bei der Indikationsstellung neu.

Lässt sich die Pulmonal- bzw. Conduitinsuffizienz mittels perkutaner Klappenimplantation beheben, wird dieser Eingriff gegenüber der Chirurgie favorisiert. Ein Klappenersatz bei schwerer Insuffizienz wird immer bei symptomatischen Patienten oder bei Abnahme der objektivierten Leistungsfähigkeit empfohlen. Weitere Indikationen sind die progressive Dilatation des rechten Ventrikels, ein enddiastolisches Volumen von 160 ml/m^2^ oder mehr respektive ein endsystolisches Volumen von 80 ml/m^2^ oder mehr, die Abnahme der systolischen Funktion oder die Zunahme der Trikuspidalinsuffizienz. Die verwendeten Volumina definieren primär die Grenzwerte, ab denen nach Klappenersatz mit keiner Normalisierung der rechtsventrikulären Dimensionen und Funktion mehr gerechnet werden kann [17]. Kritische Stimmen wenden ein, dass diese Indikation nicht auf klinischen Endpunkten beruht und zu einem unnötigen Klappenersatz mit nachteiligen Konsequenzen im Langzeitverlauf führen könnte [18]. Dennoch setzt sich die Haltung immer mehr durch, den Zeitpunkt der Klappenintervention auf Volumina abzustützen. Sie wird auch in den amerikanischen Leitlinien abgebildet [19]. Neu wird auch die Rolle des Elektrophysiologen bei der Risikostratifizierung des plötzlichen Herztodes, der Indikationsstellung zur ICD-Implantation und zur Ablation mit oder ohne geplanten Klappenersatz explizit aufgelistet.

## Transposition der großen Gefäße

**Figure 4:**
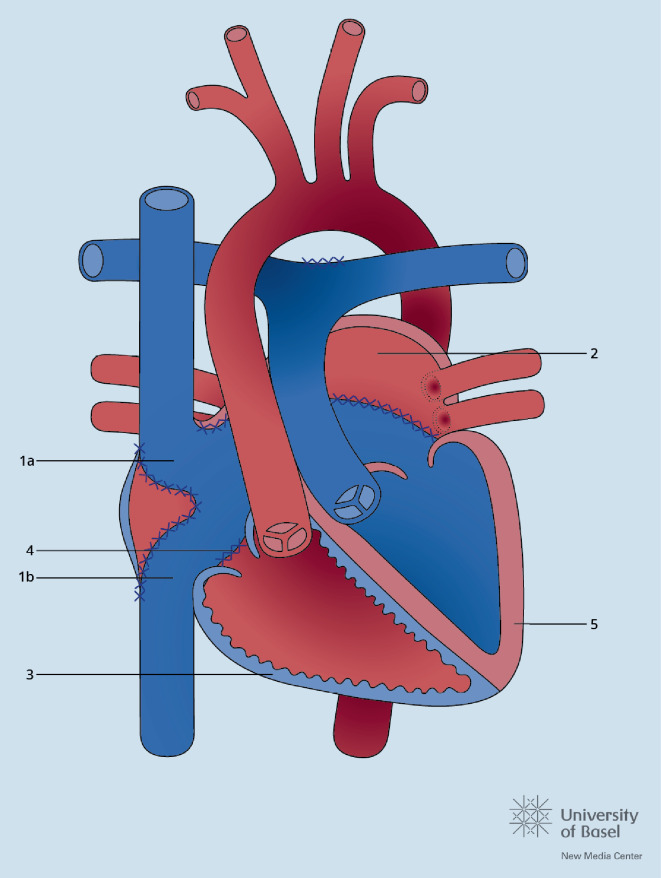

**Figure 5:**
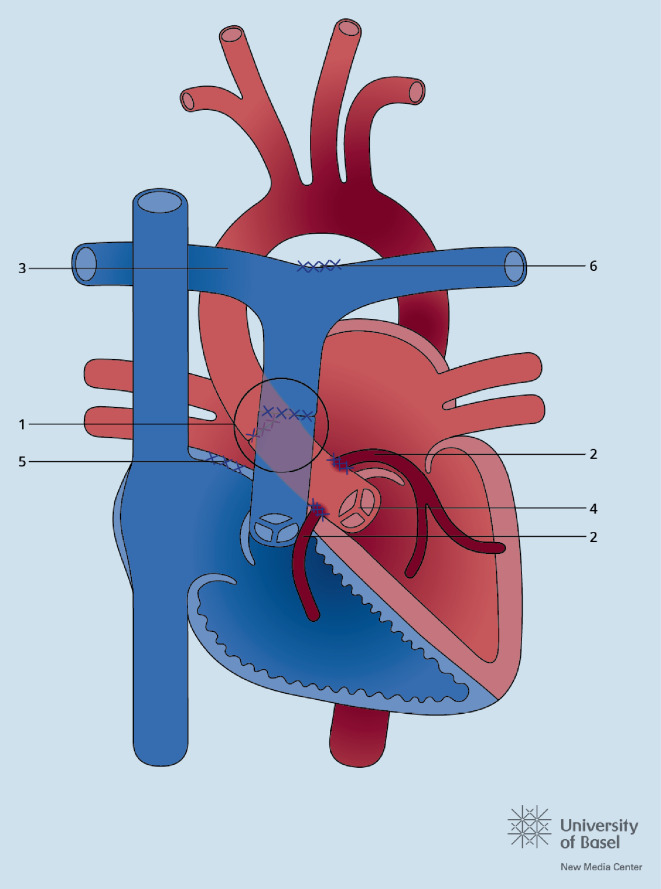

**Figure 6:**
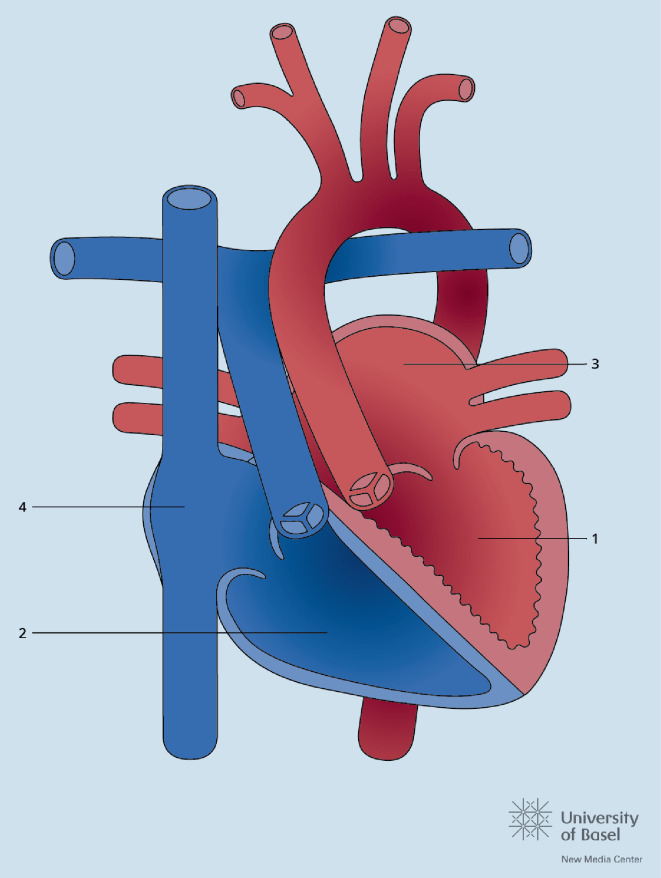

Nach der Fallot-Tetralogie ist die Transposition der großen Gefäße (TGA) der zweithäufigste zyanotische Herzfehler. Noch ist die Mehrheit der heutigen Erwachsenen mit TGA in der Kindheit mit einer Vorhofumkehr (Abb. 4) operiert worden, doch die Anzahl der jungen Erwachsenen mit einer arteriellen Switch-Operation (Abb. 5) wächst. Die Langzeitkomplikationen beider Korrekturoperationen sind verschieden. Bei den Patienten mit Vorhofumkehr stehen Arrhythmien, Komplikationen im Bereich der Venentunnels („baffles“) und die Herzinsuffizienz im Vordergrund, bei den Patienten mit arteriellem Switch die Pulmonalstenose, die Dilatation der Neoaorta und Neoaorteninsuffizienz sowie Probleme im Bereich der reimplantierten Koronarien. Eine weitere Gruppe stellen die Erwachsenen mit kongenital korrigierter TGA (ccTGA) dar, d. h. mit atrioventrikulärer und ventrikuloarterieller Diskonkordanz. Auch bei diesen Patienten, wie nach Vorhofumkehr, liegt der anatomisch rechte Ventrikel in subaortaler Position (Abb. 6). Entsprechend wird im Langzeitverlauf die Systemventrikeldysfunktion gefürchtet, hinzu kommen hier auch der komplette atrioventrikuläre (AV-)Block, die Obstruktion im subpulmonalen Ausflusstrakt und die Insuffizienz der systemischen AV-Klappe. Es wird empfohlen, sowohl nach Vorhofumkehr als auch bei ccTGA eine schwere Trikuspidalinsuffizienz nur zu beheben, sofern die Pumpfunktion des Systemventrikels höchstens leicht eingeschränkt ist (EF > 40 %). Bei der ccTGA wird generell ein Klappenersatz empfohlen. Nach Vorhofumkehr kann auch eine Rekonstruktion der Trikuspidalklappe in Betracht gezogen werden. Doch oft endet auch hier der Eingriff im Klappenersatz. Sofern mittels Kontrastechokardiographie danach gesucht wird, finden sich bei mehr als 50 % aller Patienten mit TGA und Vorhofumkehr „baffle leaks“ [20]. Insbesondere vor der Implantation eines endovenösen Schrittmacherkabels sollte ein Leck vorher verschlossen werden, da ansonsten das Risiko embolischer Ereignisse zunimmt.

Eine Herausforderung im Langzeitverlauf bei Vorhofumkehr und ccTGA bleibt die Entwicklung einer Herzinsuffizienz des Systemventrikels, sprich des morphologisch rechten Ventrikels. Diesbezüglich wurde 2016 ein Positionspapier publiziert, das die Therapiemöglichkeiten zusammenstellt [21]. In den aktuellen Leitlinien wird in dem Zusammenhang noch einmal betont, dass im Falle einer Schrittmacherimplantation aufgrund eines kompletten AV-Blocks biventrikuläres „pacing“ in Betracht gezogen werden sollte.

## Univentrikuläres Herz und Fontan-Zirkulation

Der Begriff „univentrikuläres Herz“ fasst verschiedene Defekte zusammen:

- Atresie der Trikuspidalklappe und andere Varianten des hypoplastischen rechten Herzens,
- hypoplastisches Linksherzsyndrom,
- Double-Inlet-Ventrikel,
- unbalancierter kompletter atrioventrikulärer Septumdefekt.

Ob palliativ eine Fontan-Zirkulation angelegt wird oder nicht – in allen Fällen sollte die Betreuung dieser hoch komplexen Patienten selbst bei nichtkardialen Eingriffen oder Abklärungen im spezialisierten Zentrum erfolgen.

Oft haben Patienten mit Fontan-Zirkulation in Kindheit und Jugend wenig Symptome, doch im Erwachsenenalter treten unweigerlich gesundheitliche Probleme auf, die in aller Regel dem chronisch erhöhten zentralvenösen Druck geschuldet sind. Insbesondere die „Fontan-associated liver disease“ wurde in den letzten Jahren detailliert charakterisiert. Mittlerweile sind hepatologische Verlaufskontrollen auch bei Erwachsenen mit Fontan-Zirkulation selbst bei guter Hämodynamik Teil der regulären Betreuung.

**Figure 7:**
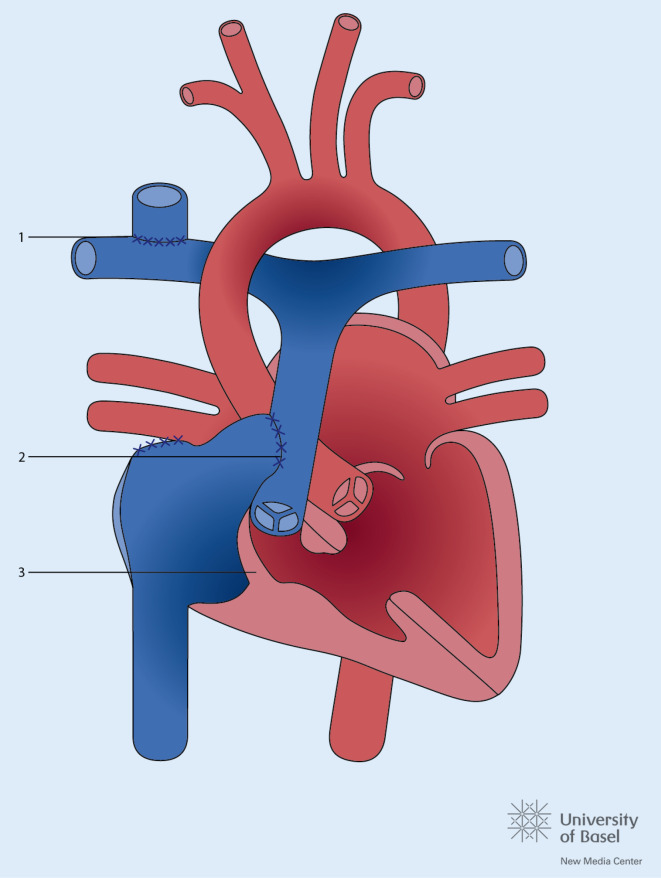

Speziell beim älteren Patienten mit klassischer Anastomose des rechten Vorhofohrs mit der Pulmonalarterie sind atriale Arrhythmien nur eine Frage der Zeit (Abb. 7). Sie können bei allen Patienten auftreten. Die Leitlinien empfehlen ein rasches Handeln, was sowohl die Elektrokonversion als auch die spätere elektrophysiologische Abklärung und Ablation betrifft. Zudem wird die Schwelle für den Beginn einer oralen Antikoagulation niedrig gehalten, wobei aufgrund fehlender Daten die Vitamin-K-Antagonisten bevorzugt eingesetzt werden sollten. Weiterhin ist die invasive Evaluation der Fontan-Zirkulation ein wichtiger Abklärungsschritt bei abnehmender Leistungsfähigkeit, neuen Arrhythmien, zunehmender Zyanose, Ödemen oder Vorliegen einer Eiweißverlustenteropathie. Pulmonale Vasoaktiva (Endothelinrezeptorantagonisten oder Phosphodiesterase-5-Inhibitoren) können eingesetzt werden, sofern eine klinische Verschlechterung durch erhöhte pulmonale Widerstände bei weiterhin normalem enddiastolischen Füllungsdruck erklärt werden kann.

## Koronaranomalien

Zum ersten Mal nehmen die Leitlinien Stellung zum Vorgehen bei bekannten oder neu diagnostizierten Koronaranomalien. Der Ursprung der linken Kranzarterie aus der Lungenarterie (als Bland-White-Garland-Syndrom oder ALCAPA [„anomalous left coronary artery from the pulmonary artery“] bezeichnet) ist immer eine Operationsindikation. Entspringt die rechte Kranzarterie der Lungenarterie, sind Symptome oder ein Ischämienachweis ebenfalls Behandlungsindikationen. Im klinischen Alltag ist der Ursprung der rechten oder linken Kranzarterie aus dem jeweils gegenüberliegenden Sinus Valsalva die häufigste Koronaranomalie. Dies sind häufig Zufallsbefunde, und es ist schwierig, den Krankheitswert dieser Koronaranomalien einzuschätzen. Zu beachten ist, dass der Verlauf der Kranzarterie beim Ursprung der rechten Kranzarterie aus dem linken Sinus Valsalva in der großen Mehrheit der Fälle interarteriell, d. h. zwischen Aorta und Pulmonalarterie, zu liegen kommt, sodass dies für sich allein kein Argument zur Korrektur ist. Generell wird das Risiko des plötzlichen Herztodes aufgrund dieser Koronaranomalien nach dem 35. Lebensjahr als gering eingestuft. Dennoch werden bestimmte anatomische Voraussetzungen (schlitzförmiges Ostium, langer intramuraler Verlauf, spitzer Abgangswinkel, Ostium >1 cm oberhalb des sinotubulären Übergangs) als prognostisch ungünstige Faktoren eingestuft. Eine chirurgische Korrektur einer Koronaranomalie, unabhängig von der Lokalisation und dem Verlauf in Bezug auf die großen Gefäße, wird allen Patienten mit myokardialer Ischämie empfohlen, wobei als Untersuchungsmethode selbst bei asymptomatischen Patienten bevorzugt eine physikalische Belastung mit Bildgebung gewählt werden sollte. Beim asymptomatischen Patienten mit abnormalem Ursprung der linken Kranzarterie und einer Risikoanatomie kann ebenfalls ein Eingriff erwogen werden, auch ohne Ischämienachweis, falls die Diagnose vor dem 35. Lebensjahr gestellt worden ist.

## Fazit für die Praxis

- Erwachsene mit angeborenem Herzfehler sind eine stetig wachsende und älter werdende Patientengruppe, die in der Mehrheit der Fälle eine spezialisierte lebenslange Betreuung benötigt. Aspekte wie Transition der Betreuung oder Advance Care Planning im späteren Leben sind Bausteine einer gesamtheitlichen Betreuung.
- Je älter die Patienten werden, desto häufiger ist mit Langzeitkomplikationen zu rechnen. Dies gilt beispielsweise für Arrhythmien. Patienten mit mäßig oder hoch komplexem Herzfehler profitieren von spezialisierten ACHD(„adult congenital heart disease“)-Zentren mit besonderer rhythmologischer Expertise. Elektrophysiologische Abklärungen, Ablation von Arrhythmien und die ICD(implantierbarer Kardioverter-Defibrillator)-Implantation spielen eine zunehmend wichtige Rolle im Therapiekonzept verschiedener Herzfehler. Insbesondere bei der korrigierten Fallot-Tetralogie kommt dies exemplarisch zum Ausdruck.
- Trotz Fortschritten im Bereich der Bildgebung ist der Herzkatheter weiterhin unentbehrlich. Die Indikationsstellung zur Intervention bei Patienten mit Aortenisthmusstenose, aber auch beim Patienten mit einem Shunt und möglicher pulmonaler Hypertonie beruht auf invasiv erhobenen Daten.
- Die Entwicklung in Bereich der perkutanen strukturellen Eingriffe hat zur Folge, dass die klassische Herzchirurgie bei bestimmten Vitien nicht mehr die primäre Therapieoption darstellt.
- Patienten mit Herzfehlern bedürfen einer defektspezifischen Behandlungsstrategie. Entsprechend der Vielzahl der Herzfehler und möglicher Korrekturoperationen in der Kindheit beruht die überwältigende Mehrheit der aktuell gültigen Empfehlungen auf der Meinung von Experten, da größere randomisierte klinische Studien fehlen bzw. nicht machbar sind. Entsprechend sind die Erfahrung des betreuenden Spezialisten sowie die Kenntnisse der jeweils gültigen Behandlungsempfehlungen wichtig.
